# VarStack^2^—an interactive web tool for somatic variant interpretation in cancer

**DOI:** 10.1093/database/baag016

**Published:** 2026-04-11

**Authors:** Nitin Sreekumar, Benjamin J Ahn, Shulan Tian, Alper Uzun, Ece D Gamsiz Uzun

**Affiliations:** Department of Pathology and Laboratory Medicine, The Warren Alpert Medical School of Brown University, 593 Eddy Street, Providence, RI 02903, United States; The Warren Alpert Medical School of Brown University, 222 Richmond Street, Providence, RI 02912, United States; Division of Computational Biology, Mayo Clinic, Department of Quantitative Health Sciences, 200 First St. SW, Rochester, MN 55905, United States; Department of Pathology and Laboratory Medicine, The Warren Alpert Medical School of Brown University, 593 Eddy Street, Providence, RI 02903, United States; Legorreta Cancer Center, Brown University, 70 Ship Street Providence, RI, Providence, RI 02903, United States; Brown Center for Clinical Cancer Informatics and Data Science (CCIDS), 593 Eddy Street, Providence, RI 02903, United States; Center for Computational Molecular Biology (CCMB), 164 Angell Street, Providence, RI 02912, United States; Department of Pathology and Laboratory Medicine, The Warren Alpert Medical School of Brown University, 593 Eddy Street, Providence, RI 02903, United States; Legorreta Cancer Center, Brown University, 70 Ship Street Providence, RI, Providence, RI 02903, United States; Brown Center for Clinical Cancer Informatics and Data Science (CCIDS), 593 Eddy Street, Providence, RI 02903, United States; Center for Computational Molecular Biology (CCMB), 164 Angell Street, Providence, RI 02912, United States; Department Pathology and Laboratory Medicine, Brown University Health, 593 Eddy Street, Providence, RI 02903, United States

## Abstract

The rapid progress in tumour genome sequencing has created a need for bioinformatics tools to interpret the clinical significance of detected variants. VarStack² integrates information from several publicly available resources, including the Catalogue of Somatic Mutations in Cancer (COSMIC), ClinVar, cBioPortal, UCSC Genome Browser, and ClinicalTrials.gov, CIViC and presents it through a user-friendly interface. VarStack^2^ simplifies the process of retrieving data, saving users significant time compared to manually navigating each database individually. Users can input a variant by specifying a gene symbol, amino acid change, and coding sequence change, with the option to search tumour-specific studies in cBioPortal alongside their primary query. Results are organized into separate sections and can be exported in CSV format for further analysis. Additionally, VarStack^2^ offers a smart search feature that suggests variants for the gene of interest based on its database search results. These features make VarStack^2^ a useful tool for scientists and clinicians by enhancing the variant interpretation process and integrating somatic variant information into workflows. VarStack² is freely available at http://varstack.brown.edu/

## Introduction

Cancer is a complex disease and the second leading cause of mortality worldwide. Many distinct causes, including mutations in specific genes, have been linked to the development of cancer [1]. The advances in next-generation sequencing (NGS) technologies have fostered the growth of available genomic data, such as those from DNA sequencing (DNA-seq), RNA sequencing (RNA-seq), and chromatin immunoprecipitation sequencing (ChIP-seq). Several publicly available databases, including Catalogue of Somatic Mutations in Cancer (COSMIC) [2], ClinVar [3], cBioPortal [4], and ClinicalTrials.gov [5], provide genomic and clinical data from patients across different tumour types.

COSMIC provides information regarding somatic mutations reported in cancer. As of December 2025, COSMIC includes information on 29 million somatic mutations linked to human cancers [2]. ClinVar provides clinical significance information on somatic and germline variants [3]. cBioPortal facilitates easy interpretation and visualization of genomic variations reported in multiple studies, including The Cancer Genome Atlas (TCGA) and the Cancer Cell Line Encyclopedia (CCLE) datasets. The UCSC genome browser is a useful tool to visualize the genome over several annotation tracks, including variants [6, 7]. ClinicalTrials.gov is a database that compiles ongoing and completed clinical trials [5]. Clinical Interpretation of Variants in Cancer (CIViC) is an open-source platform that allows users to provide their expert input on variants [8]. VariantAlert is a web-based tool to provide updates in the variant annotations in other databases such as ClinVar and COSMIC [9]. The Drug–Gene Interaction Database (DGIdb) is a publicly available resource for therapeutic search based on genes [10]. VarSome is another tool used for variant classification [11]. We also developed a web tool, Variant Graph Craft [12], to visualize variant calling files (VCFs), providing information from gnomAD [13] and ClinVar. Effective use of genomic data will support research endeavours in cancer precision medicine, leading to improved health outcomes [14]. Variant interpretation often requires an individual search of each tool and database, which can be time-consuming. There is a need for the development of a variant interpretation tool that gathers and organizes the data from individual databases, with the advantage of significantly decreasing the time spent using each database and tool individually. We developed VarStack in 2020, a web tool providing somatic variant information from COSMIC, cBioPortal, ClinVar, and UCSC Genome Browser via a single search [15]. VarStack required data to be uploaded to the server intermittently. Moreover, while all the data from online databases are returned to the website, the data from each database is displayed in separate tabs. We updated the web tool and developed VarStack^2^, which now uses application programming interfaces (APIs) to retrieve information from databases in real time and displays the results in a one-page format. VarStack^2^ features a smart search which was not included in VarStack.

## Methods

### Architecture

VarStack^2^ is built using the ReactJS framework, which facilitates extendable design schemes, making it simple to add new databases to the site if updates are needed. Each data parsing function takes the imputed gene name, amino acid, and coding sequence change as an input, and returns the appropriate HTML to render the data. Data from COSMIC, cBioPortal, ClinicalTrials.gov, and CIViC are retrieved in real-time by using APIs for each database. For the UCSC Genome Browser, an iframe is used to visualize the genome by entering genomic coordinates. Information from ClinVar is also provided using iframe. The smart search feature, which enables users to retrieve existing variants for a given gene, is implemented through API calls to the COSMIC database.

## Results

### Running VarStack^2^

**Figure 1:**
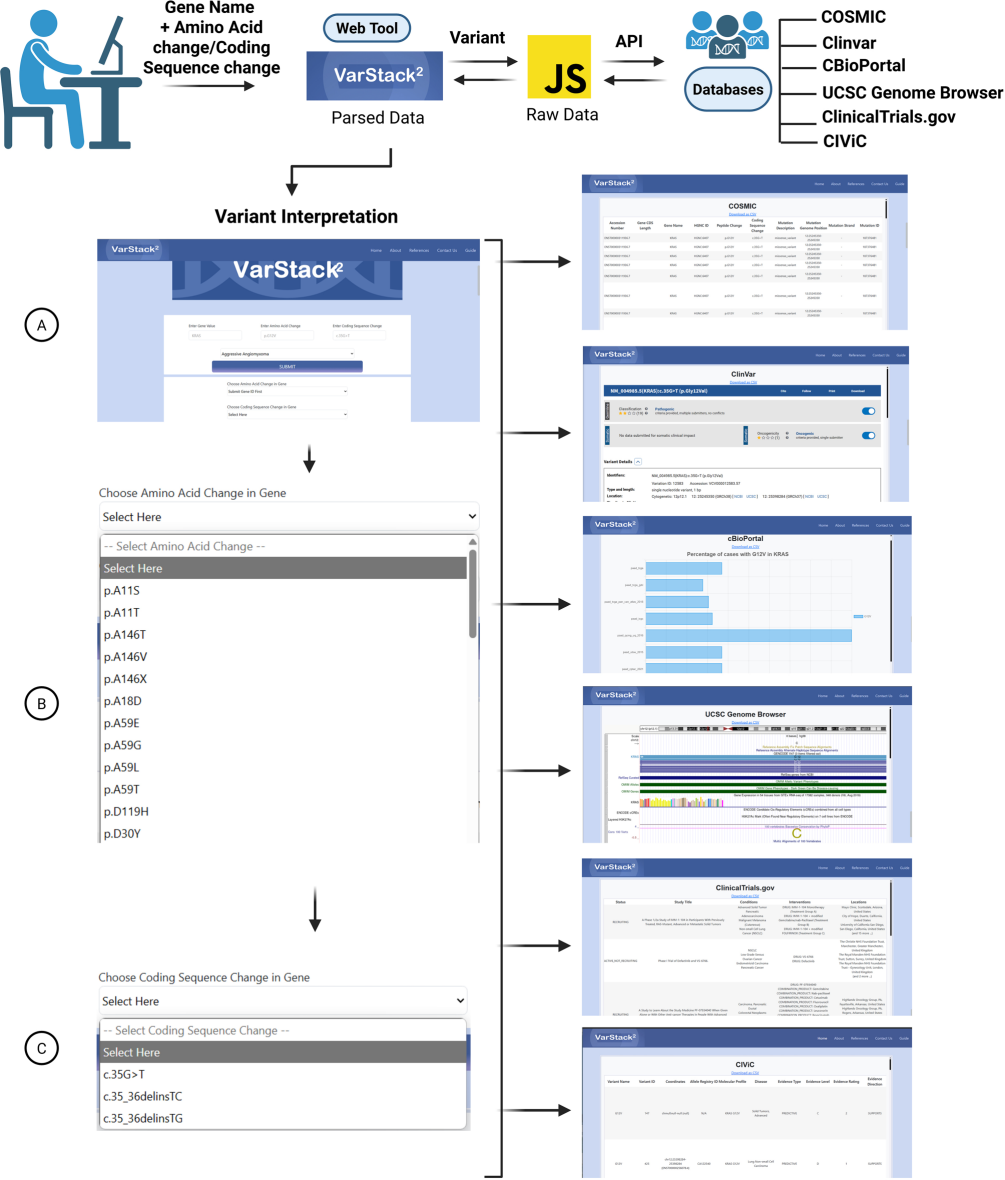
VarStack^2^ uses APIs to retrieve information from COSMIC, ClinVar, cBioPortal, UCSC Genome Browser, ClinicalTrials.gov, and CIViC. An iframe was used for UCSC Genome Browser and ClinVar.

VarStack^2^ is an updated version of VarStack with additional features (Fig. 1). It retrieves somatic variant information from COSMIC, ClinVar, cBioPortal, the UCSC Genome Browser, and ClinicalTrials.gov as well as CIViC, and displays the information on one page in separate sections for each database. VarStack^2^ takes the gene name, amino acid change, and nucleotide change as input. It also allows the users to input tumour type to explore cBioPortal data. VarStack^2^ implements a smart search that automatically lists the variants in the selected gene. The ClinVar section allows the users to view the information directly from the website. The cBioPortal section includes a frequency plot for the variant of interest in the selected tumour type. The UCSC Genome Browser section allows the users to visualize the genome without leaving the page. The ClinicalTrials.gov section provides information about clinical trials on the selected variant. The CIViC section provides information on the clinical significance of the selected variants. The variant data visualized on the web tool can be saved as a CSV file for further analysis. A guide with step-by-step instructions is provided on the website for the users.

### Case study

**Figure 2:**
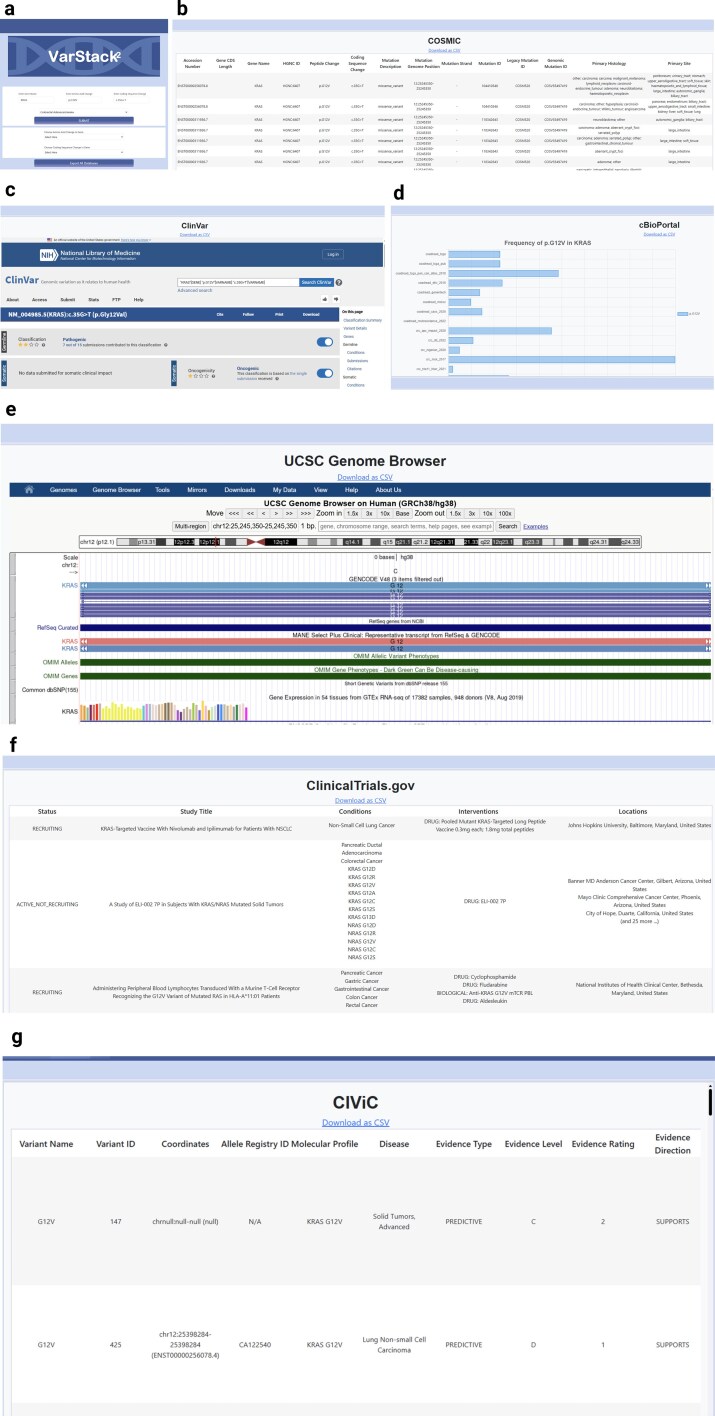
Case study to interpret KRAS p.G12V in Colorectal Adenocarcinoma using VarStack^2^. The variant and tumour type are entered in search window (a) VarStack^2^ retrieves information from (b) COSMIC, (c) ClinVar, (d) cBioPortal, (e) UCSC Genome Browser, and (f) ClinicalTrials.gov, (g) CIViC and displays in separate windows.

This case study demonstrates how VarStack^2^ can be used to interpret KRAS p.G12V in Colorectal Adenocarcinoma (Fig. 2).

### Step 1: enter the variant in VarStack^2^

On the VarStack^2^ web tool, enter ‘*KRAS*’ under gene, ‘p.G12V’ under amino acid change, and ‘c.35G > T’ under coding sequence change. In the dropdown menu, select ‘Colorectal Adenocarcinoma’ and submit (Fig. 2a).

### Step 2: review information on the variant

Upon submission, VarStack^2^ retrieves data from multiple databases, displaying results in separate windows for COSMIC, ClinVar, cBioPortal, UCSC Genome Browser, CIViC, and ClinicalTrials.gov:

**COSMIC**: This section lists all COSMIC entries for the selected variant, providing detailed mutation information (Fig. 2b).

**ClinVar**: This section displays ClinVar classification, oncogenicity, HGVS annotation, and previously reported cases. In this example, ClinVar identifies KRAS p.G12V as a pathogenic variant in an oncogenic gene (Fig. 2c).

**cBioPortal**: This section presents a bar plot of the variant frequency across multiple studies for the selected tumour type (Fig. 2d).

**UCSC Genome Browser**: Users can visualize the variant within the *KRAS* gene, along with transcript details and additional tracks (e.g. OMIM, dbSNP, and RepeatMasker). Track visibility can be customized by the users on the window (Fig. 2e).

**ClinicalTrials.gov**: This section lists all relevant clinical trials, including those recruiting, not recruiting, or completed for the selected variant (Fig. 2f). Links to each trial were provided for further information.

**CIViC:** This section shows expert-curated information on the clinical significance of variants (Fig. 2g).

This structured workflow presented in this case study allows users to efficiently gather and analyse key variant data from multiple sources.

## Discussion

VarStack^2^ is a web tool that allows users to search for specific mutations and aggregates information from COSMIC, ClinVar, cBioPortal, UCSC Genome Browser, ClinicalTrials.gov, and CIViC. Using real-time API calls, it presents the variant information in a single-page, user-friendly interface with smart search and CSV export features. While other integrative resources (e.g. VarSome, OncoKB, MyCancerGenome, ClinVar Miner, Ensembl VEP, and cBioPortal) focus on curation, algorithmic classification, or cohort-level analysis, they often require programming skills, multiple queries, or commercial access (Table 1). In contrast, VarStack^2^ streamlines variant information across tumour types, allowing scientists and clinicians to access multiple databases quickly and efficiently, reducing the time needed for interpretation.

**Table 1:** Comparison of functionality, data sources, and usability between VarStack2 and existing variant interpretation platforms

Feature	VarStack^2^	OncoKB	CIViC	MyCancerGenome	ClinVar miner	Ensembl VEP	DGIdb	cBioPortal	VarSome
Sources covered	COSMIC ClinVar cBioPortal UCSC Genome Browser CIViC ClinicalTrials.gov	Expert-curated	Expert-curated	Expert-curated	ClinVar data	Ensembl	Various sources for drug–gene interaction search	Genomic data from 500+ studies	140 databases for variant classification
Licensing	Public	Free/ Commercial	Public	Public	Public	Public	Public	Public	Free/ commercial
Export (CSV/VCF)	CSV	Limited	Limited	Limited	Limited	Limited	Limited	Limited	Limited
Batch processing	Yes	No	No	No	No	Limited	Limited	Limited	Limited
VCF support	No	No	No	No	No	Yes	No	No	Yes
Audit trail/sources traceable	Yes	Partial	Yes	Partial	Yes	Partial	Partial	Partial	Yes

Advances in NGS and bioinformatics methods have drastically increased the amount of genomic data, leading to improved precision medicine [16]. Web tools such as VarStack^2^ are needed for efficient analysis of genomic data. NGS has become an important component of clinical testing requiring efficient and sensitive variant interpretation with reasonable turn-around time. Searching multiple databases is a time-consuming process for the researchers and clinicians who may review several variants in a day. Although there are commercially available tools for variant interpretation with knowledge-based databases, those require funds. Thus, there is still a need for a publicly available web tool that can retrieve variant information from multiple databases and display the results on one page. By providing the up-to-date variant information with an easy-to-use interface, VarStack^2^ reduces the time needed for variant interpretation.

## Conclusion

VarStack^2^ is designed to assist scientists and clinicians in interpreting somatic variants across multiple tumour types. Navigating various databases for several samples can be time-consuming, and some databases require bioinformatics or programming expertise. VarStack^2^’s user-friendly interface eliminates the need for such specialized knowledge and saves time. The sections in the user interface display data from multiple databases in a one-page format, offering smart search and downloadable file options for practical use.
